# Impacts of Sublethal Doses of Spinetoram on the Biological Traits and Detoxifying Enzymes of the Tomato Leaf Miner, *Tuta absoluta* (Lepidoptera: Gelechiidae)

**DOI:** 10.3390/insects15120990

**Published:** 2024-12-13

**Authors:** Mingjun Jiang, Xiujuan Qian, Zhaoxu Zhou, Yueying Liu, Meijiao Zhang, Yaxian Yang

**Affiliations:** 1 College of Plant Protection, Gansu Agricultural University, Lanzhou 730000, China; 18009378360@163.com (M.J.);; 2 Plant Protection Research Institute, Gansu Academy of Agricultural Sciences, Lanzhou 730000, China; 3 Gansu Province Agricultural Pest Natural Enemy Engineering Research Center, Lanzhou 730000, China

**Keywords:** spinetoram, *Tuta absoluta*, survival and developmental, antioxidant enzymes, detoxifying enzymes

## Abstract

*Tuta absoluta* is a major pest, damaging tomato crops in many countries. Spinetoram, a novel insecticide, is increasingly used for the management of various insect pests. However, limited information is available on its lethal effects on *T. absoluta*. Therefore, this study was carried out to evaluate the toxicity of spinetoram concentrations in *T. absoluta* by exposing third-instar larvae to this insecticide through leaf-dipping bioassays under laboratory conditions. The results showed that sublethal spinetoram concentrations (LC_10_ and LC_20_) extended the developmental duration of the larval and pupal stage, as well as the oviposition period, and decreased the pupal weight, pupation and eclosion rates, fecundity, sex ratio, and egg-hatching rate compared to the control group. LC_10_ and LC_20_ treatments up-regulated the activity of superoxide dismutase and catalase antioxidant enzymes in *T. absoluta* larvae by 51.14–88.72% and 39.34–87.76% at 12 h, 62.71–96.68% and 37.38–39.92% at 24 h, and 41.78–53.34% and 19.44–48.39% at 48 h, respectively, compared to the control. Conversely, the LC_10_ and LC_20_ treatments inhibited the activities of detoxifying enzymes, i.e., carboxylesterase (14.72–34.95%), acetylcholinesterase (9.02–48.86%), glutathione S-transferase (19.40–43.19%), and mixed-function oxidase (18.18–25.93%). These findings indicate that lethal concentrations of spinetoram influence the population dynamics of *T. absoluta* by reducing their survival and developmental potential and inhibiting the activity of detoxifying enzymes.

## Simple Summary

*Tuta absoluta* is one of the most prevalent pests in tomato crops, and efficient control measures are essential to reduce damage to tomato crops. Here, the toxicity effects of spinetoram, a novel insecticide, against *T. absoluta* were evaluated in laboratory bioassays. This study examined the sublethal concentrations (LC_10_ and LC_20_) of spinetoram on the biological features of *T. absoluta*, as well as the activity of detoxification and antioxidant enzymes. Our results provide evidence that spinetoram negatively impacts *T. absoluta* by reducing their survival, developmental potential, and enzyme activity, thereby affecting pest population dynamics. Therefore, spinetoram insecticide can be used as a potential chemical strategy for controlling the *T. absoluta* population in tomato crops.

## 1. Introduction

The tomato (*Lycopersicon esculentum*) is globally a major cultivated crop, daily consumed as a fruit and vegetable in fresh and processed form due to its versatile nutritional benefits [1,2]. It is widely acknowledged as a powerhouse of nutrients, offering essential minerals, sugars, vitamins, antioxidants, and dietary fibers. In particular, lycopene, an antioxidant found in tomatoes, has gained attention for its potential medicinal benefits and anti-cancer properties [3]. Due to their minimal environmental requirements for growth, tomatoes are extensively cultivated worldwide and represent the most economically important vegetable crop. Globally, tomatoes are cultivated over an area of 5 million hectares [4]. According to FAO statistics, about 68.24 million tons of tomatoes were produced in China during 2022 [5], accounting for about 16.7% of the global total production. With the growing awareness of its health-related benefits and an increase in the global population, the demand for its cultivation has increased tremendously in recent years. In addition, tomato cultivation supports the livelihoods of millions of farmers and plays a substantial role in the global food economy [1]. Despite its widespread cultivation and recognized health benefits, tomato production faces significant agricultural challenges, particularly from pests and diseases that compromise both yields and fruit quality, resulting in economic losses [1].

Among the myriad of insect pest attacks that afflict tomato crops, *Tuta absoluta* (Meyrick) (Lepidoptera: Gelechiidae), is one of the most prevalent and damaging pests [6,7,8]. It is commonly known as the tomato leaf miner, distinguished by its erratic feeding behavior targeting the leaves, stems, and fruit of tomato plants [4]. It has been reported that over 87% of global tomato production is directly threatened by *T. absoluta* infestation, causing huge environmental and economic losses [7,9]. Typically, the females lay eggs on the undersides of the leaves or stems. After hatching, the larvae damage the plants’ foliage by feeding on the mesophyll tissues, causing necrosis and the wilting of leaves over time [10], leading to reduced photosynthetic efficiency and, ultimately, the decreased yield and quality of tomatoes and their market value [9]. A previous study indicated that this pest can cause a potential yield reduction of up to 90% [7]. In severe cases, losses in tomato production can reach 100% if appropriate management measures are not implemented [4]. In addition, *T. absoluta* has a rapid life cycle, with a generation duration of approximately 25 to 30 days under optimal conditions, allowing multiple generations within each crop growing season [6,10]. Such a rapid reproductive character makes this insect more dangerous and notorious, necessitating efficient pest management for a sustainable tomato farming system.

Spinetoram is a semi-synthetic chemical that belongs to the novel spinosyn group (Group 5, IRAC mode of action classification) of insecticides [11,12]. Spinetoram acts as a nicotinic acetylcholine receptor (nAChR) agonist, and its primary mode of action involves disrupting neurotransmission in insects, resulting in paralysis and, ultimately, mortality [8,13]. Spinetoram exhibits high efficacy against a wide range of insect pests while exhibiting minimal effects on the majority of beneficial insect populations [14,15,16,17]. In recent years, there has been growing attention to the sublethal impacts of insecticides on insect populations. The toxicity of pesticide application in agricultural fields decreases gradually over time as a result of natural environmental processes [18]. This exposure may lead to insects being poisoned at a non-lethal level, displaying distinct signs, referred to as a sublethal concentration [19]. Sublethal doses can disrupt the population dynamics across multiple generations of insects, leading to various sublethal effects [20,21]. Besides their immediate lethal effects, insecticides may also have prolonged effects on the targeted pests. Therefore, it is imperative to evaluate both the sublethal and lethal impacts of insecticides in pest control management. Integrating sublethal effects into pest management measures can improve the efficacy of pest population reduction and promote the long-term sustainability of agricultural systems [18]. The extensive and prolonged use of chemical insecticides may cause the development of resistance, thereby compromising the efficacy of control measures [8,22,23]. The development of resistance to insecticides is closely linked to the sublethal effects of the insecticide on insect populations, leading to changes in endogenous antioxidant and detoxification enzyme systems [24,25]. These enzymes are capable of metabolizing various toxins in insects to maintain their physiological functions. Superoxide dismutase (SOD) and catalase (CAT) are the primary antioxidant enzymes that effectively eliminate excessive reactive oxygen species (ROS) in insects and maintain oxidative balance [26]. Detoxifying enzymes include carboxylesterase (CarE), acetylcholinesterase (AChE), glutathione S-transferase (GST), and mixed-function oxidase (MFO), which play crucial roles in the development of insecticide resistance [27,28]. These enzymes are induced by various endogenous and exogenous factors, enabling the rapid adaptation of insects to insecticides and other environmental stresses [16,29]. Previous studies have indicated that insecticides have varying effects on insect defense systems, with alterations in enzyme activity correlating to insect mortality or the development of resistance [8,12,14,18,24]. Therefore, analyzing changes in antioxidant and detoxification enzymes is essential for investigating the sublethal doses of insecticides in insect toxicology.

Despite the extensive research on the efficacy of spinosyn insecticides against numerous insect species [14,23,25,30,31], there is still a lack of information regarding the effectiveness of spinetoram and its sublethal concentrations against *T. absoluta*. Therefore, the present study aims to examine the effects of different concentrations of spinetoram on survival, growth behavior, reproduction, and the activities of antioxidant and detoxifying enzymes in *T. absoluta*. The research findings are anticipated to contribute to the growing body of knowledge aimed at developing effective strategies for the mitigation and pest management of *T. absoluta*.

## 2. Materials and Methods

### 2.1. Collection of T. absoluta

The larvae of the leaf miner were collected in November 2023 from Suzhou District in Jiuquan City (98°20′ E, 39° N). The collected larvae were raised in the laboratory, without any exposure to pesticides. The rearing temperature for the larvae was 25 ± 1 °C, with a relative humidity level of 40–70% and a light–darkness cycle of 16 and 8 h, respectively. Tomato leaves were supplied for larvae feeding. The adult leaf miners were then raised in an insect-rearing cage, supplemented with cotton soaked in a 10% honey solution to enhance their nutritional intake.

### 2.2. Assessment of Acute Toxicity

The acute toxicity of spinetoram in tomato leaf miner larvae was determined following the immersion method. Spinetoram with 92% active ingredient purity, was purchased from Dow AgroSciences and used in the experiment. The stock solution was prepared by dissolving the spinetoram in acetone to achieve a concentration of 0.1 g L^−1^. Later, the stock solution was diluted with distilled water to form the desired six concentration gradients (0.1 mg L^−1^, 0.2 mg L^−1^, 0.4 mg L^−1^, 0.8 mg L^−1^, 1.6 mg L^−1^, and 3.2 mg L^−1^) based on the preliminary experiments. In addition, acetone was used as a control treatment. Tomato leaves of uniform size (12–14 cm long and 5–6 cm wide) were immersed in these solutions for 10 **s** and then allowed to dry naturally. Twenty larvae were subsequently placed in each sterile Petri dish. Four replications were used for each treatment. The Petri dishes were covered with plastic wrap, punctured to allow aeration, and kept in an incubator at a temperature of 25 ± 1 °C, with a relative humidity of 40–70% and a light/dark cycle of 16/8 h.

After 48 h, the mortality rate of the third-instar larvae of the tomato leaf miner was observed and recorded after each treatment. Larvae were classified as dead if no movement was observed or they displayed a blackened and rigid appearance. Data were analyzed with SPSS 21.0 software (IBM Co., Ltd., Armonk, NY, USA) using the probit link function to develop a toxicity regression equation for spinetoram in the third-instar larvae of the tomato leaf miner. Subsequently, a regression equation was employed to determine the lethal concentrations of spinetoram (Table 1).

### 2.3. Treatment with Sublethal Spinetoram Concentrations

Based on the toxicity assay, concentrations of spinetoram corresponding to LC_10_ and LC_20_ were prepared. Following the leaf-dipping method, the third-instar larvae of the leaf miner were exposed to sublethal spinetoram concentrations (LC_10_ and LC_20_) and the control. The larvae were subsequently placed on the tomato leaves and transferred to sterile Petri dishes. Each treatment consisted of three repeats, with 80 larvae in each repeat. The Petri dishes were kept at a temperature of 25 ± 1 °C, relative humidity of 70 + 5%, and light/dark cycle of 16/8 h.

After 48 h, the healthy and surviving larvae from each treatment were selected and individually transferred to untreated leaves in separate disposable Petri dishes. Fresh leaves were regularly substituted, and daily observations were conducted to record the survival and development of the larvae until they reached the pupal stage.

The weights of the male and female pupae were measured, and they were subsequently transferred to 50 mL centrifuge tubes. The duration of the larval development stage and cumulative pupation rates were calculated.

$$Cumulative\;pupation\;rate=\frac{No.of\;pupae\;on\;ith\;day+No.of\;pupated\;larvae}{total\;number\;of\;larvae\;}\times 100$$

Newly emerged male and female adults were paired individually in 50 mL centrifuge tubes, which contained cotton soaked in 10% honey water and fresh leaves. Daily observations were conducted to record the rate of egg hatching and the number of eggs laid by the adults. During the experiment, a 10% honey-water solution and tomato leaves were regularly replenished to support the normal development of both the tomato leaf miner larvae and the adults.

### 2.4. Assay of Detoxification Enzymes

The third-instar larvae were collected after 12, 24, and 48 h of exposure to sublethal concentrations of spinetoram along with the control group for the determination of detoxification enzyme activities (acetylcholinesterase, carboxylesterase, glutathione S-transferase, and mixed-function oxidase). The assay samples were prepared following previous methods [18]. The midguts of the larvae were extracted with dissecting tweezers and transferred into sterile centrifuge tubes containing 1 mL of pre-cooled 0.1 mM sodium phosphate buffer solution (pH 7.0). The samples were subsequently centrifuged (12,000× *g*) at 4 °C for 10 min. The supernatant was collected as the source of enzymes and placed on ice before testing. The supernatants were used to determine the activity of four detoxification enzymes using commercial assay kits (Jianglaibio Co., Ltd., Shanghai, China), following the manufacturer’s instructions.

Superoxide dismutase (SOD) activity was determined using NBT (nitro blue tetrazolium) photo-reduction, while catalase (CAT) activity was estimated following the H_2_O_2_ decomposition method [27].

### 2.5. Statistical Analysis

Data were analyzed using one-way ANOVA (analysis of variance) and post-hoc multiple comparisons (Tukey’s test) to determine significant differences in the growth attributes in *T. absoluta* larvae among the different insecticide treatments. In addition, a two-way ANOVA was performed to determine the effects of the insecticide treatments, sampling intervals, and their interactive effect on the detoxification and antioxidant enzymes. A probit analysis was used to estimate the lethal and sublethal concentrations of spinetoram. The log-probit regression model was employed to estimate the lethal concentrations (LC_10_, LC_20_, and LC_50_) and the slopes of the regression lines using SPSS software version 21.0 (IBM Co., Ltd., Armonk, NY, USA). Figures were generated using Excel 2010 (Microsoft Corp., Redmond, WA, USA) and GraphPad Prism 8.0 (GraphPad Software, San Diego, CA, USA).

## 3. Results

### 3.1. Assessment of the Toxicity Test of Spinetoram

The acute toxicity of spinetoram to the larvae of tomato leaf miners is presented in Table 1. Following 48 h of insecticide exposure, the mortality rates with the spinetoram concentrations (0.1, 0.2, 0.4, 0.8, 1.6, and 3.2 mg L^−1^) were significantly (*p* < 0.05) higher than those of the control group. The median lethal concentration (LC_50_) after 48 h of treatment was determined to be 0.32 mg L^−1^, with sublethal doses of LC_10_ and LC_20_ recorded at 0.06 mg L^−1^ and 0.10 mg L^−1^, respectively (Table 1). The dose–response line of spinetoram was Y = 1.69x + 0.84, with a relative coefficient of 0.95 (R^2^).

### 3.2. Spinetoram Sublethal Effect on the Biological Characteristics of T. absoluta

**Figure 1:**
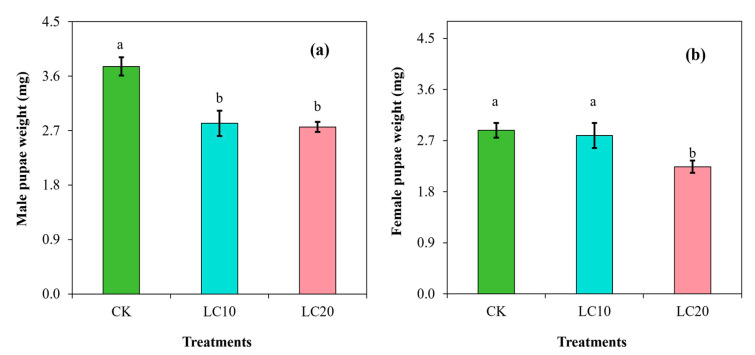
Effects of spinetoram concentrations (LC_10_ and LC_20_) on the weight of the male pupae (**a**) and female pupae (**b**) of *T. absoluta*. Different lowercase letters indicate significant differences among treatments following Tukey’s test at *p* < 0.01.

Figure 1 demonstrates the impact of the sublethal doses of spinetoram on the pupal weight of *T. absoluta*. The results indicated a dose-dependent effect of spinetoram on male pupae, resulting in a significant reduction (F = 45.29; df = 3; *p* < 0.0001) in pupal weight (Figure 1a). The weight of the male pupae subjected to LC_10_ and LC_20_ treatments decreased by 25.01% and 26.60%, respectively, compared to the control group. The effects of spinetoram on female pupal weight showed no significant difference between LC_10_ and the control group (Figure 1b). However, the LC_20_ treatment resulted in a 22.23% decrease in the weight of female pupae compared to the control (F = 24.26; df = 3; *p* < 0.001).

**Figure 2:**
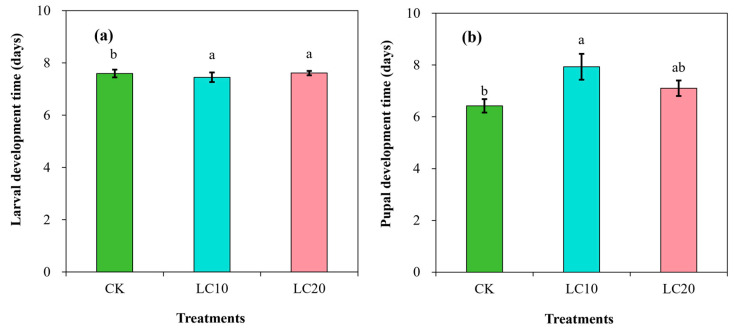
Effects of spinetoram concentrations (LC_10_ and LC_20_) on the development of the larvae (**a**) and pupae (**b**) of *T. absoluta*. Different lowercase letters indicate significant differences among treatments following Tukey’s test at *p* < 0.01.

The results also indicated significant effects of spinetoram treatments on the duration of larval (F = 22.66; df = 3; *p* < 0.001) and pupal (F = 22.11; df = 3; *p* < 0.01) development stages (Figure 2). The mean duration of larval development in the control group was 6.6 days. However, the LC_10_ (7.5 days) and LC_20_ (7.6 days) treatments significantly prolonged the larval stage (Figure 2a). In addition, the duration of pupal development in the control group was 6.4 days. This duration was significantly prolonged by LC_10_ treatment (7.9 days), while LC_20_ treatment resulted in a slight reduction in the period to 7.1 days (Figure 2b).

**Figure 3:**
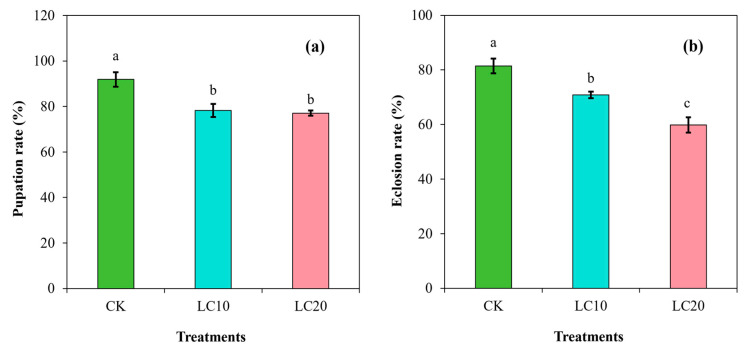
Effects of spinetoram concentrations (LC_10_ and LC_20_) on the pupation rate (**a**) and eclosion rate (**b**). Different lowercase letters indicate significant differences among treatments following Tukey’s test at *p* < 0.01.

In addition, the pupation rate (F = 45.29; df = 3; *p* < 0.0001) and eclosion rate (F = 45.29; df = 3; *p* < 0.0001) were significantly affected by the spinetoram concentrations (Figure 3). The mean pupation rate in the control group was 91.88%, which decreased to 78.34% with the LC_10_ treatment and to 77.13% with the LC_20_ treatment (Figure 3a). Moreover, the mean eclosion rate in the control group was 81.41%, which was significantly reduced by the LC_10_ treatment (70.85%) and the LC_20_ treatment (59.81%) (Figure 3b). In comparison, the LC_10_ and LC_20_ treatments decreased the pupation rate by 14.74% and 16.05%, while the eclosion rates were reduced by 12.97% and 26.53%, respectively, compared to the control group.

**Figure 4:**
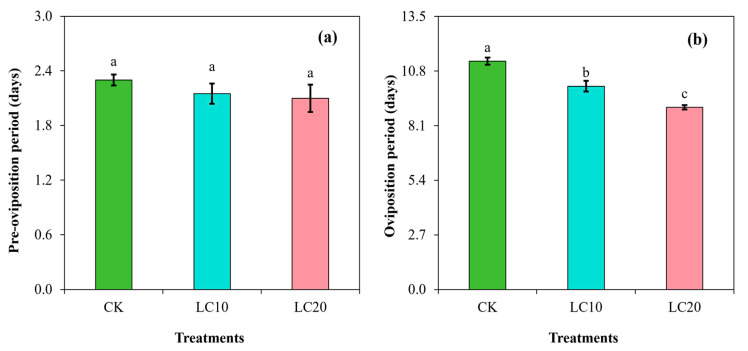
Effects of spinetoram concentrations (LC_10_ and LC_20_) on the pre-oviposition period (**a**) and oviposition period (**b**) of *T. absoluta*. Different lowercase letters indicate significant differences among treatments following Tukey’s test at *p* < 0.01.

The results demonstrated no significant effect (F = 1.72; df = 3; *p* = 2572) of insecticide treatments on the pre-oviposition period when compared to the control group (Figure 4a). However, the LC_10_ (10.1 days) and LC_20_ (9.0 days) treatments significantly (F = 113.6; df = 3; *p* < 0.0001) decreased the oviposition period in comparison to the control group (11.3 days) (Figure 4b).

**Figure 5:**
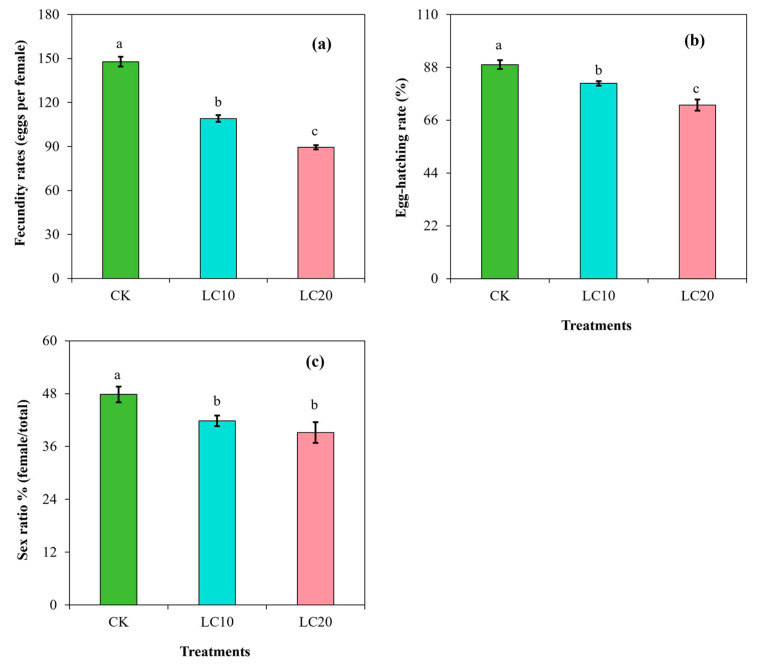
Effects of spinetoram concentrations (LC_10_ and LC_20_) on the fecundity rate (**a**), egg-hatching rate (**b**), and sex ratio (**c**) of *T. absoluta*. Different lowercase letters indicate significant differences among treatments following Tukey’s test at *p* < 0.01.

The fecundity rate (F = 523.55; df = 3; *p* < 0.0001) and the egg-hatching rate (F = 182.99; df = 3; *p* < 0.0001) were also significantly affected by the insecticide treatment. The fecundity rate and egg-hatching rate were higher in the control group and exhibited a decreasing trend with the application of spinetoram in a dose-dependent manner (Figure 5). The LC_10_ and LC_20_ treatments resulted in a decrease in the egg numbers laid per female by 25.87% and 39.53% (Figure 5a), while the egg-hatching rate was inhibited by 8.75% and 18.88%, respectively, compared to the control group (Figure 5b).

In addition, the sex ratio expressed in percentage (female/total) differed significantly between the control group and the insecticide treatment groups (F = 17.48; df = 3; *p* < 0.01). The sex ratio was higher in the control group (47.8%) than in the LC_10_ (41.8%) and LC_20_ (39.2%) treatment groups. In comparison, the LC_10_ and LC_20_ treatments decreased the sex ratio by 12.55% and 17.99%, respectively, compared to the control (Figure 5c).

### 3.3. Spinetoram Sublethal Effects on the Detoxification Enzymes of T. absoluta Larvae

**Figure 6:**
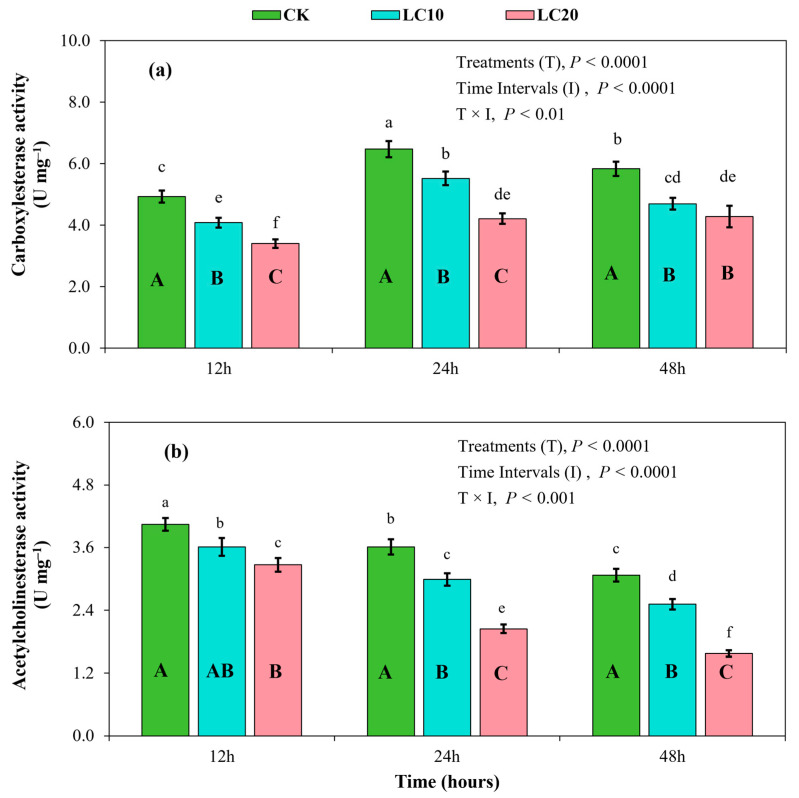
Effects of spinetoram concentrations (LC_10_ and LC_20_) on carboxylesterase activity (**a**) and acetylcholinesterase activity (**b**) in *T. absoluta* larvae. Different lowercase letters indicate significant differences among all spinetoram treatments across all sampling intervals at *p* < 0.01. Different uppercase letters indicate significant differences among the treatments at each sampling interval at *p* < 0.01.

**Figure 7:**
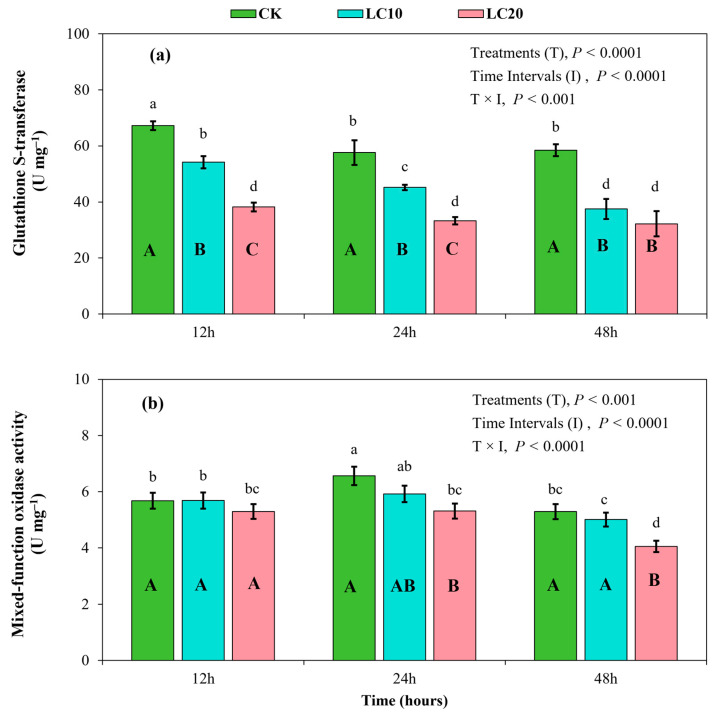
Effects of spinetoram concentrations (LC_10_ and LC_20_) on glutathione S-transferase activity (**a**) and mixed-function oxidase activity (**b**) in *T. absoluta* larvae. Different lowercase letters indicate significant differences among all treatments across all sampling intervals at *p* < 0.01. Different uppercase letters indicate significant differences among treatments at each sampling interval at *p* < 0.01.

The spinetoram treatments (F = 197.20; df = 2; *p* < 0.0001), sampling intervals (F = 101.58; df = 2; *p* < 0.0001), and their interaction (F = 5.46; df = 4; *p* < 0.01) significantly affected carboxylesterase activity in the *T. absoluta* larvae (Figure 6 and Figure 7). Carboxylesterase activity exhibited an initial increase from 12 h to 24 h, followed by a slight decrease at 48 h (Figure 6a). At each sampling interval (12 h, 24 h, and 48 h), enzyme activity in the *T. absoluta* larvae was significantly lower than in the control group. Carboxylesterase activity was inhibited by the LC_10_ and LC_20_ treatments, exhibiting a decrease in activity by 17.22% and 30.99% at 12 h, 14.72% and 34.95% at 24 h, and 19.49% and 26.67% at 48 h, respectively, compared to the control group (Figure 6a).

The spinetoram treatments (F = 361.46; df = 2; *p* < 0.0001), sampling intervals (F = 350.93; df = 2; *p* < 0.0001), and their interaction (F = 15.60; df = 4; *p* < 0.001) significantly affected acetylcholinesterase activity in the *T. absoluta* larvae (Figure 6b). The results indicated a gradual decline in acetylcholinesterase activity over time after exposure to the treatments, with the lowest values observed at 48 h across all treatments. At each sampling interval (12 h, 24 h, and 48 h), enzyme activity in the test insects in the control group was the highest (Figure 6b). However, enzyme activity was markedly inhibited by the insecticide concentration treatments. Enzyme activity with the LC_10_ and LC_20_ treatments decreased by 9.02% and 18.04% at 12 h, 18.08% and 44.11% at 24 h, and 18.24% and 48.86% at 48 h, respectively, compared to the control group.

In addition, the activity of glutathione S-transferase was significantly affected by the spinetoram treatments (F = 396.55; df = 2; *p* < 0.0001), sampling intervals (F = 66.17; df = 2; *p* < 0.0001), and their interaction (F = 7.10; df = 4; *p* < 0.001). Glutathione S-transferase activity exhibited a declining trend over time after exposure to the insecticide treatments (Figure 7a). Enzyme activity peaked at 12 h (38.19–67.22 U mg^−1^) and decreased to lower levels at 48 h (32.20–58.48 U mg^−1^). The LC_10_ and LC_20_ treatments resulted in a reduction in glutathione S-transferase activity compared to the control group, depicting a decrease of 19.40% and 43.19% at 12 h, 21.56% and 42.17% at 24 h, and 35.84% and 39.81% at 48 h, respectively (Figure 7a).

Furthermore, mixed-function oxidase activity in the *T. absoluta* larvae was significantly affected by the spinetoram treatments (F = 53.26; df = 2; *p* < 0.001), sampling intervals (F = 76.04; df = 2; *p* < 0.0001), and their interaction (F = 5.28; df = 4; *p* < 0.01) (Figure 7b). At 12 h, no significant difference was observed among the LC_10_ treatment, the LC_20_ treatment, and the control group. At 24 h and 48 h, enzyme activity was significantly inhibited by the LC_20_ treatment, whereas enzyme activity with the LC_10_ treatment did not show a significant difference compared to the control group (Figure 7b). In comparison, the LC_20_ treatment decreased mixed-function oxidase activity by 18.18% and 25.93% at 24 h and 48 h, respectively, compared to the control.

### 3.4. Spinetoram Sublethal Effect on Antioxidant Enzymes in T. absoluta Larvae

**Figure 8:**
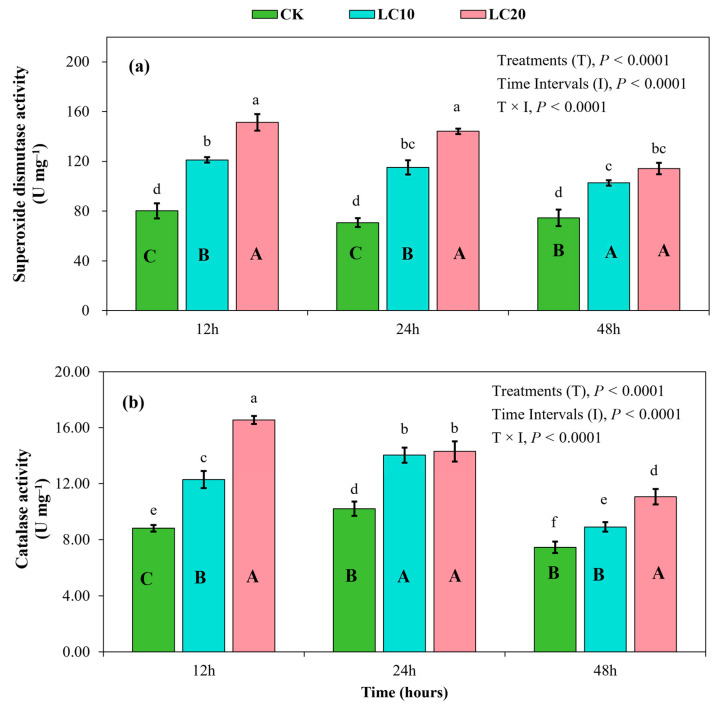
Effects of the spinetoram concentrations (LC_10_ and LC_20_) on superoxide dismutase (SOD) activity (**a**) and catalase (CAT) activity (**b**) in *T. absoluta* larvae. Different lowercase letters indicate significant differences among all treatments across all sampling intervals at *p* < 0.01. Different uppercase letters indicate significant differences among the treatments at each sampling interval at *p* < 0.01.

Results from the present study depicted significant effects of spinetoram concentrations (F = 400.09; df = 2; *p* < 0.0001), sampling intervals (F = 40.96; df = 2; *p* < 0.0001), and their interaction (F = 11.44; df = 4; *p* < 0.0001) on SOD activity in *T. absoluta* larvae (Figure 8). SOD activity followed a gradually declining trend over time after exposure to insecticide stress (Figure 8a). SOD activity in the larvae ranged from 80.21 to 151.37 U mg^−1^ at 12 h, from 70.80 to 139.25 U mg^−1^ at 24 h, and from 74.56 to 114.33 U mg^−1^ at 48 h. The spinetoram treatments resulted in an upregulation of SOD activity, with the LC_20_ treatment exhibiting greater activity than both the control and the LC_10_ treatment at 12 h and 24 h. Nevertheless, no significant difference was observed between the LC_10_ and LC_20_ treatments at 48 h (Figure 8a). Compared to the control, enzyme activity under the LC_10_ and LC_20_ treatments increased by 51.14% and 88.72% at 12 h, 62.71% and 96.68% at 24 h, and 41.78% and 53.34% at 48 h, respectively.

CAT activity in the *T. absoluta* larvae was also significantly affected by the spinetoram concentrations (F = 255.72; df = 2; *p* < 0.0001), sampling intervals (F = 135.81; df = 2; *p* < 0.0001), and their interaction (F = 23.71; df = 4; *p* < 0.0001). CAT activity exhibited a decreasing trend over time following exposure to spinetoram treatments (Figure 8b). CAT activity was observed to be higher at 12 h (8.82–16.56 U mg^−1^) but comparatively lower at 48 h (7.46–11.07 U mg^−1^). CAT activity increased following insecticide exposure, with the highest levels observed in the LC_20_ treatment group. The LC_10_ and LC_20_ treatments increased CAT activity by 39.34% and 87.75% at 12 h, 37.38% and 39.92% at 24 h, and 19.44% and 48.39% at 48 h, respectively, compared to the control group.

## 4. Discussion

Spinosyn insecticides, such as spinosad [23,25] and spinetoram [31,32], are recognized to have great efficacy against several insect pests. Their applications are reported to cause lethal (mortality) and sublethal effects; therefore, both effects should be considered while assessing the overall impact of these insecticides [22,33]. According to the LC_50_ values presented in Table 1, spinetoram demonstrated significant efficacy against *T. absoluta*. These results are consistent with the findings of previous studies reporting the high toxicity of spinetoram against *Frankliniella occidentalis* [34], *Helicoverpa armigera* [32], and *Spodoptera frugiperda* [35]. Rabea et al. [36] reported that spinosad caused the greatest toxicity toward *Tetranychus urticae*, with an LC_50_ value of 6.72 mg L^−1^. The LC_50_ values of the 96S and 96-1Ac strains of *H. armigera* were reported as 1.30 and 0.62 mg kg^−1^ at 24 h and 0.84 and 0.39 mg kg^−1^ at 72 h, respectively [32]. Our results indicated an LC_50_ value of 0.32 mg L^−1^ for *T. absoluta*, suggesting better toxicity. The toxic effects of spinosyn insecticides involves disrupting the insect nervous system [15], causing involuntary muscle contractions and paralysis, and ultimately resulting in death [13]. Moreover, the enhanced sensitivity to the insecticide could be associated with various biochemical and physiological mechanisms, such as the modulation of detoxification enzyme activity. Previous studies have highlighted that exposure to insecticides significantly decreases detoxification enzyme activity, which plays a role in inhibiting metabolic activity, leading to acute toxicity in insects [25,27].

Besides acute toxicity, insecticide application can have several sublethal effects on insect pests, influenced by the difference in concentrations and changes in pesticide efficacy over time after application [23,25]. Sublethal effects refer to the behavioral or physiological impacts on insects that endure following exposure to a pesticide [30]. Behavioral effects may impact feeding, foraging ability, and oviposition [17]. Physiological changes can result in a decrease in the development rate, lifespan, fertility, and fecundity, as well as alterations to the sex ratio [14,25,37]. Previously, the sublethal effects of spinosyn insecticides have been examined in various insect pests. For instance, the sublethal concentrations of spinosad exposure negatively impacted the development and survival of larvae, reduced larval weight, decreased the pupation ratio and pupal weight, and decreased the emergence ratio and fecundity of *H. armigera* [25]. Spinetoram has been found to reduce the rate of pupa formation, the adult longevity, and the adult emergence of *H. didymator* [38]. Sublethal concentrations of spinetoram prolonged the total longevity, adult pre-ovipositional period, and total pre-ovipositional period while decreasing preadult survival in *S. frugiperda* [35]. Wei et al. [32] observed that sublethal doses of spinetoram disrupted the developmental duration of the larval stage in *H. armigera*. The findings from the present study also demonstrated the significant effects of sublethal concentrations of spinosad on the behavioral and physiological traits of *T. absoluta*. Spinetoram treatments resulted in the reduced weight of male and female pupae, prolonged the development duration of the larval and pupal stages, and reduced both the pupation and eclosion rates of *T. absoluta*. The impact of spinosad on biological characteristics was more pronounced with the LC_20_ treatment than with the LC_10_ treatment. The observed effects can be attributed to interference by the chemical treatments of the key metabolic processes or disruption of the hormonal pathways essential for development. These results align with previous study findings that demonstrated a dose-dependent developmental impact of spinosad on *P. xylostella* [23], *H. armigera* [30], *H. armigera* [32], and *S. frugiperda* [35].

Studies have shown that sublethal concentrations of different pesticides can also impact the fecundity of target insects [20,25]. In the present study, sublethal concentrations of spinetoram were found to be effective by disrupting the longevity period and reducing the sex ratio, fecundity rates (eggs per female), and egg-hatching rate of *T. absoluta*. These results are in agreement with previous findings in which spinosad treatment resulted in a decrease in fecundity and egg size, along with the lower hatchability of smaller eggs in *P. xylostella* [23]. In addition, previous studies have highlighted that spinosyn application negatively influences the developmental duration and fecundity, as well as the reproductive behavior, feeding behavior, body weight, and fertility, of different insect pests [25,39]. Our findings also indicated that spinetoram concentrations (LC_10_ and LC_20_) negatively affected the developmental process and duration of *T. absoluta*, influencing the pre-oviposition duration, oviposition duration, and fecundity. All these parameters followed a steady decline with the increase in spinetoram concentration. The decrease in fecundity with insecticide treatments is attributed to the physiological and morphological alterations in both the male and female species. In this study, the results demonstrated a significant decrease in the male and female pupal weight of *T. absoluta* following spinetoram treatments at LC_10_ and LC_20_ concentrations. The egg-hatching rate with the LC_10_ and LC_20_ treatments was significantly reduced compared to the control group, which is in agreement with the findings of previous studies [23,35].

Insect pests can acquire tolerance to insecticide exposure and may develop resistance over time by modulating the activities of specific endogenous detoxification enzymes, reflecting a biochemical adaptation strategy to chemical stress [12,23,40]. Various insecticides have been reported to either up-regulate or down-regulate the activity of detoxifying enzymes in insects, thereby contributing to the evolution of insect resistance [14,39,41]. Previous studies have demonstrated a significant relationship between insecticide resistance and high levels of detoxifying enzymes in different insects [18,42]. The AchE enzyme catalyzes the degradation of acetylcholine, a neurotransmitter within the central nervous system of insects, making it a common target for neurotoxic agents [43]. The present study demonstrated a significant inhibition of AchE enzyme activity in *T. absoluta* larvae after exposure to spinetoram treatments, with LC_20_ resulting in the greatest inhibition capacity. Different doses of the same insecticide may have varying effects on the activity of detoxification enzymes in insect species [14,26]. The inhibitory effect of spinetoram on AchE activity may have resulted from modifications to the active site, potentially causing a substantial accumulation of acetylcholine at synapses, thereby inhibiting neural signal transmission, as reported in previous studies [30,44]. CarE primarily facilitates the degradation of toxins, hormones, and pheromones, thereby mediating neurodevelopmental functions [45]. The results of this study indicate a gradual decline in CarE activity following exposure to spinetoram treatments. CarE activity was markedly lower with spinetoram treatments in a dose-dependent manner, with LC_20_ exhibiting the lowest values compared to the control group. Previously, Hu et. al. [26] reported that spinosyn insecticide interacts with the CarE enzyme by inhibiting the conversion of ester compounds into alcohols and acids. This represents a potential mechanism underlying spinetoram-induced toxicity in *T. absoluta* larvae. GST is another important detoxifying enzyme in insects [18]. In the present study, spinetoram treatments also inhibited the activity of the GST enzyme in *T. absoluta* larvae. The observed inhibition may have resulted from reduced nucleophilic conjugation reactions between endogenous glutathione and exogenous electrophilic substances after exposure to spinetoram [14]. Furthermore, high concentrations of spinetoram (LC_20_ treatment) inhibited MFO activity, while the effect of LC_10_ was less pronounced compared to the control group. These results suggest that the spinetoram insecticide can be used as a potential inhibitor of detoxifying enzymes, and hence, for the effective control of the *T. absoluta* pest.

In addition to detoxifying enzymes, antioxidant enzymes (specifically SOD and CAT) play an important role in the oxidative stress responses of insects [26]. SOD catalyzes the conversion of superoxide anion radicals into H_2_O_2_, which is subsequently decomposed into water and oxygen through the action of CAT [46]. The results of the present study indicated a significant upregulation of SOD and CAT enzyme activity in *T. absoluta* larvae. The elevation of SOD and CAT activity reflects the adaptive response to oxidative stress induced by insecticides. These results are in agreement with those of previous studies, indicating that insecticides have significant impacts on the activity of antioxidant enzymes across different insect species. For instance, Chen et. [26] reported that a sublethal concentration (LC_20_) of the carvacrol insecticide activated the activities of SOD and CAT enzymes in *L. dispar* at 12 h, 24 h, and 48 h after chemical exposure. A similar trend in the increase of antioxidant enzymes with insecticide exposure was also reported in *L. dispar* by another study [46]. Previous studies have proposed that the upregulation of antioxidants is associated with insecticide resistance [27,47]. Our results suggest that although an increase in antioxidant enzymes represents an adaptive strategy to mitigate oxidative stress, it may not cause resistance in *T. absoluta* due to the significant inhibition of primary detoxifying enzymes (CarE, AChE, GST, and MFO) with the application of spinetoram.

## 5. Conclusions

In conclusion, the results of this study indicate that spinetoram has both lethal and sublethal effects, which may negatively impact the growth and development dynamics of *T. absoluta*. The concentration of spinetoram (LC_10_ and LC_20_) decreased the pupal weight, pupation and eclosion rates, female fecundity, and sex ratio (female/total), which will potentially impact the population density of subsequent generations. The delay in the developmental duration of larval and pupal stages, as well as the pre-oviposition and oviposition periods, may affect the timing and duration of pest occurrence. Furthermore, spinetoram treatments increased the activity of antioxidant enzymes but markedly inhibited the activities of detoxifying enzymes compared to the control group. Hence, spinetoram application offers promising avenues for the effective management of the *T. absoluta* pest. However, the complexities of pest and environmental factors may impact the efficacy of pesticides and necessitate a careful and informed approach. Therefore, additional field trials are recommended to better evaluate the long-term effects of spinetoram application on *T. absoluta* populations and potential resistance.

## Figures and Tables

**Table 1:** Lethal and sublethal concentrations of spinetoram against the *T. absoluta* larvae.

Insecticide	Concentrations (mg L^−1^)			Goodness of Fit		
	LC_10_ (95% CI) ^a^	LC_20_ (95% CI)	LC_50_ (95% CI)	Slope ± SE ^b^	χ^2^ (df) ^c^	*p*
Spinetoram	0.06 (0.03–0.08)	0.10 (0.06–0.14)	0.32 (0.24–0.41)	1.69 ± 0.20	1.6 (6)	0.95

LC_10_, LC_20_, and LC_50_ denote the concentrations of spinetoram that are lethal to 10%, 20%, and 50% of *T. absoluta*, respectively. ^a^ 95% CI represents 95% confident intervals. ^b^ Standard error ^c^ Chi-square value (χ^2^) and degrees of freedom (df).

## Data Availability

The data can be made available upon request.
